# Conhecimento sobre Atividade Física e seus Níveis em Crianças com Cardiopatia Congênita

**DOI:** 10.36660/abc.20200286

**Published:** 2020-05-22

**Authors:** Adilson Marques, Bruna Gouveia

**Affiliations:** 1 CIPER Faculdade de Motricidade Humana Universidade de Lisboa Lisboa Portugal CIPER, Faculdade de Motricidade Humana, Universidade de Lisboa, Lisboa - Portugal; 2 ISAMB Faculdade de Medicina Universidade de Lisboa Lisboa Portugal ISAMB, Faculdade de Medicina, Universidade de Lisboa, Lisboa - Portugal; 3 Instituto de Administração da Saúde IP-RAM Ilha da Madeira Portugal Instituto de Administração da Saúde, IP-RAM, Ilha da Madeira - Portugal; 4 Interactive Technologies Institute LARSyS Lisboa Portugal Interactive Technologies Institute, LARSyS, Lisboa – Portugal

**Keywords:** Cardiopatias Congênitas, Comorbidade, Crianças, Adolescentes, Exercício, Atividade Física, Conhecimento, Condicionamento Físico, Qualidade de Vida

Os benefícios da atividade física já estão bem documentados. Em crianças e adolescentes, a atividade física melhora a saúde cardiovascular, óssea e metabólica, níveis de condicionamento físico, peso corporal e condição do sono.^1^ Além disso, há evidências dos benefícios cognitivos, psicológicos e sociais da atividade física.^1^ Os benefícios da atividade física sobre a saúde são transversais a todas as crianças, incluindo aquelas que vivem com uma doença crônica, como doença cardíaca congênita (DCC), prevenindo comorbidades e melhorando a qualidade de vida.^2,3^

A importância da atividade física para crianças com DCC já foi documentada há muito tempo.^4^ A participação em atividades físicas é de grande importância para as crianças com DCC, pois elas correm o risco de desenvolver outras doenças cardiovasculares e metabólicas,^3,5^ sinais crescentes de depressão e ansiedade.6 Além disso, quando presentes, esses problemas de saúde podem ser mitigados por um estilo de vida ativo.^1-3^

Embora os benefícios da atividade física para a saúde sejam bem reconhecidos, as evidências mostram que algumas crianças com DCC não praticam atividade física suficiente para atingir os níveis recomendados,^7^ e a atividade física diminui e os comportamentos sedentários aumentam com a idade em ambos os sexos.^5^ Além disso, os níveis de atividade física de crianças com DCC são inferiores aos de crianças saudáveis.^8^Portanto, é importante identificar os fatores associados a níveis menores de atividade física.

Apesar da importância da atividade física para a saúde das crianças com DCC, a mesma não é considerada como tendo um valor-alvo.^9^ Como a maioria dos pais concentra sua atenção no desempenho acadêmico em direção a uma carreira desejada, a atividade física parece ser menos importante. Assim, o tempo disponível é utilizado para trabalhos escolares. Além disso, os pais pensam que a atividade física não é importante em comparação com a ampla gama de outras atividades disponíveis para crianças com DCC.^9^Para vários pais, a atividade física tem um papel limitado no processo de reabilitação, de modo que eles tendem a superproteger seus filhos contra algumas práticas de atividade física.^9,10^ Além disso, supõe-se que as crianças com DCC sejam responsáveis por se envolver em comportamentos de redução de risco, e a atividade física é considerada um risco potencial para algumas pessoas. Essas concepções contribuem para os discursos individualistas do “*healthism*” (salutarismo)^10^ e crianças e pais concluem que a atividade física pode ser importante, mas não é tão importante, pois pode comprometer o desempenho acadêmico ou o estado de saúde. À luz dessas crenças, seria interessante analisar o conhecimento das crianças com DCC sobre atividade física.

Campos et al.,^11^ realizaram um estudo com o objetivo de identificar os níveis de conhecimento de crianças e adolescentes com DCC sobre sua doença e analisar a associação entre os níveis de conhecimento e a prática de atividade física. É um estudo interessante realizado em uma amostra cuidadosamente selecionada de crianças e adolescentes com DCC. Os dados foram auto-relatados, mas para este estudo em particular, o auto-relato foi o método mais apropriado para avaliar o conhecimento e os níveis de atividade física das crianças e dos adolescentes. A partir dos resultados, observou-se que muitas crianças e adolescentes tiveram dificuldades em descrever sua doença. Quase metade das crianças e adolescentes não sabia o nome do seu defeito cardíaco e apenas 24% localizou corretamente as lesões em um diagrama cardíaco. Esses resultados devem ser motivo de preocupação, porque, sem o conhecimento correto do problema de saúde, muitas ações contraproducentes podem ser tomadas. Em relação à atividade física, as crianças e adolescentes mais ativos apresentaram maior conhecimento sobre a doença. O desenho do estudo não permitiu compreender a associação entre conhecimento e prática de atividade física. No entanto, os autores fornecem algumas explicações potenciais e razoáveis. Talvez, os pais de crianças ou adolescentes que praticam atividade física estejam preocupados com os efeitos da atividade física na saúde de seus filhos. Por esse motivo, eles consultam os médicos para obter mais informações sobre atividade física e DCC. Como resultado, ao questionar as limitações da atividade física, pais e filhos ou adolescentes obtêm mais informações sobre a doença e compreendem os efeitos na saúde da atividade física. O desenho do estudo não permitiu compreender a associação entre conhecimento e prática de atividade física. No entanto, os autores fornecem algumas explicações potenciais e razoáveis. Talvez, os pais de crianças ou adolescentes que praticam atividade física estejam preocupados com os efeitos da atividade física na saúde de seus filhos. Por esse motivo, eles consultam os médicos para obter mais informações sobre atividade física e DCC. Como resultado, ao questionar as limitações da atividade física, pais e crianças ou adolescentes obtêm mais informações sobre a doença e compreendem os efeitos da atividade física na saúde. Consequentemente, crianças e adolescentes com mais conhecimento se sentem mais seguros em praticar atividades físicas regularmente. Os resultados da associação entre os níveis de conhecimento e de atividade física são parcialmente apoiados por investigações anteriores.12 É evidente que o conhecimento é importante e apoia o processo de tomada de decisão.

Modelos de comunicação e mudanças comportamentais sugerem que o conhecimento sobre um comportamento desempenha um papel significativo em convencer as pessoas a mudarem seus hábitos, e o conhecimento é necessário para que as pessoas tomem decisões sobre a saúde.^13^ Portanto, pode-se supor que o conhecimento das diretrizes de atividade física possa ser um passo em direção à mudança comportamental, com relação à adoção e/ou manutenção de um estilo de vida ativo. Estudos corroboram essa premissa, mostrando que o conhecimento da atividade física relacionada à saúde está associado ao aumento da atividade física em crianças, adolescentes e jovens.^14^ Os resultados do presente estudo^11^ e de outros realizados anteriormente^12,14^ destacam a importância de programas educacionais para aumentar o conhecimento sobre saúde. O envio da mensagem de recomendação de atividade física, especialmente entre os jovens, pode aumentar os níveis de atividade física.

Entretanto, pesquisas desenvolvidas em uma variedade de pacientes15 sugerem que o fornecimento de conhecimento, materiais e apoio profissional não é suficiente para que os pacientes realizem mudanças em relação a comportamentos saudáveis. Portanto, estratégias alternativas devem ser consideradas. Estratégias baseadas no automonitoramento de comportamentos, comunicação de riscos e uso de apoio social parecem ser as mais eficazes para mudanças comportamentais.

As evidências atuais sugerem que as recomendações de atividade física para crianças com DCC foram amplamente implementadas e foram dados conselhos de médicos e profissionais de saúde sobre os possíveis benefícios da atividade física para a saúde em pessoas com DCC, incluindo crianças. Além disso, e talvez o mais importante, isso também significa que a mensagem sobre a importância da atividade física3 foi bem aceita. Isso é importante porque, em crianças com DCC, a atividade física não está relacionada a um risco aumentado de eventos adversos, e restrições específicas se aplicam apenas a situações com problemas médicos específicos.^3^

Para crianças e adolescentes com DCC, a atividade física é ainda mais importante devido à diminuição dos níveis de aptidão física que geralmente ocorrem devido ao tempo que eles podem precisar permanecer no hospital. A atividade física, principalmente de intensidade moderada a vigorosa, está independentemente associada a melhor qualidade de vida, melhor condicionamento físico e melhor composição corporal em crianças com DCC.^1-3^
