# Supplementary material for: Conhecimento sobre Atividade Física e seus Níveis em Crianças com Cardiopatia Congênita
Source: Arq Bras Cardiol. 2020 May 22;114(5):793–4. [Article in Portuguese] doi: 10.36660/abc.20200286 (PMC8386996; doi:10.36660/abc.20200286)
Supplement: Translation

# Translation: Physical Activity Knowledge and Levels among Children with Congenital Heart Disease

**Authors:** Adilson Marques, Bruna Gouveia

**Affiliations:** 1 Portugal CIPER, Faculdade de Motricidade Humana, Universidade de Lisboa, Lisboa - Portugal; 2 Portugal ISAMB, Faculdade de Medicina, Universidade de Lisboa, Lisboa - Portugal; 3 Portugal Instituto de Administração da Saúde, IP-RAM, Ilha da Madeira - Portugal; 4 Portugal Interactive Technologies Institute, LARSyS, Lisboa – Portugal

**Keywords:** Heart Defects, Congenital, Comorbidity, Children, Adolescents, Exercise/ Physical Activity, Knowledge, Physical Conditioning, Quality of Life

The benefits of physical activity are well documented. Among children and adolescents, physical activity improves cardiovascular, bone and metabolic health, fitness levels, weight status, and sleep.^1^ Also, there is evidence of the cognitive, psychological and social benefits of physical activity.^1^The health benefits of physical activity are transversal to all children, including those living with a chronic disease, such as congenital heart disease (CHD), preventing comorbidities, while improving quality of life.^2,3^

The importance of physical activity for children with CHD has been documented for a long time.^4^ Engagement in physical activity is of high importance for children with CHD because they are at risk of developing other cardiovascular and metabolic diseases,^3,5^ increasing signs of depression and anxiety.^6^ In addition, when present, these health problems can be mitigated by an active lifestyle.^1-3^

Although the health benefits of physical activity are well recognized, evidence shows that some children with CHD do not practice enough physical activity to achieve the recommended levels,^7^ and activity declines and sedentary behaviors increase with age in both genders.^5^ Furthermore, levels of physical activity of children with CHD are lower than those in healthy children.^8^ Therefore, it is important to identify the factors associated with lower levels of physical activity.

Despite the importance of physical activity for the health of children with CHD, physical activity is not considered a target value.^9^ Because most parents put their attention on academic achievement towards a desired career, physical activity seems to be less important. Thus, the available time is used for schoolwork. Additionally, parents think that physical activity is not meaningful in comparison to the broad range of other activities that are available for children with CHD.^9^For several parents, physical activity has a limited role in the rehabilitation process, so they tend to overprotect their children against some physical activity practices.^9,10^ Besides that, it is assumed that children with CHD are responsible for engaging in risk-reducing behaviors, and physical activity is considered a potential risk for some people. These conceptions contribute to the individualist discourses of healthism,^10^ and children and parents assume that physical activity might be important, but it is not so important since it can jeopardize the academic achievement or the health status. In light of these beliefs, it would be interesting to analyze the knowledge of children with CHD about physical activity.

Campos et al.^11^ performed a study aimed at identifying the levels of knowledge of children and adolescents with CHD about their disease and to analyze the association between the levels of knowledge and the practice of physical activity. It is an interesting study performed in a carefully selected sample of children and adolescents with CHD. Data were self-reported, but for this particular study, self-report was the most appropriate method for assessing the children’s and the adolescents’ physical activity knowledge and levels. From the results, it was observed that many children and adolescents had difficulties describing their disease. Almost half of the children and adolescents did not know the name of their heart defect, and only 24% correctly located the lesions on a heart diagram. These results should be a matter of concern because, without the correct knowledge of the health problem, many counterproductive actions can be taken. Regarding the physical activity, the most active children and adolescents showed greater knowledge about the disease. The study design did not allow an understanding of the association between knowledge and physical activity practice. Nonetheless, the authors provide some potential and reasonable explanations. Perhaps, the parents of children or adolescents who enjoy physical activity are concerned about the effects of physical activity on their children’s health. For this reason, they consult doctors for more information about physical activity and CHD. As a result, by questioning the limitations of physical activity, parents and children or adolescents get more information about the disease and understand the health effects of physical activity. Consequently, children and adolescents with more knowledge feel safer to engage in physical activity regularly. The results of the association between knowledge and physical activity levels are partly supported by previous investigations.^12^ It is clear that knowledge is important and supports the decision-making process.

Models of communication and changing behaviors suggest that knowledge about a behavior plays a significant role in persuading people to change their habits, and knowledge is required for people to make health decisions.^13^ Therefore, it can be conjectured that knowledge of physical activity guidelines can be a step towards behavioral change, with respect to adopting and/or maintaining an active lifestyle. Studies have supported this assumption, showing that knowledge of health-related physical activity is linked with increased physical activity among children, adolescents, and young adults.^14^ The results of the present study,^11^ and other previously performed studies,^12,14^ highlight the importance of educational programs to increase knowledge about health. Providing the physical activity recommendation message, especially among young people, might increase the physical activity levels.

However, research developed in a variety of patients^15^suggests that providing knowledge, materials and professional support is not sufficient for patients to accomplish changes regarding healthy behaviors. Therefore, alternative strategies should be considered. Strategies based on self-monitoring of behaviors, risk communication and the use of social support seem to be the most effective for behavioral changes.

Present evidence suggest that physical activity recommendations for children with CHD have been widely implemented and medical doctors’ and health professionals’ advice have been given regarding the potential health benefits of physical activity for people with CHD, including children. Moreover, and perhaps the most important, this also means that the message of the importance of physical activity^3^ has been well accepted. This is important because, among children with CHD, physical activity is not related to an increased risk of adverse events, and particular restrictions only apply to situations with specific medical issues.^3^

For children and adolescents with CHD, physical activity is even more important due to the decreased levels of physical fitness that often occur because of the time they might have to spend at the hospital. Physical activity, mainly from moderate to vigorous intensity, is independently associated with better quality of life, improved physical fitness, and better body composition in children with CHD.^1-3^
